# Modulation of the Structure of the Conjugated Polymer TMP and the Effect of Its Structure on the Catalytic Performance of TMP–TiO_2_ under Visible Light: Catalyst Preparation, Performance and Mechanism

**DOI:** 10.3390/ma16041563

**Published:** 2023-02-13

**Authors:** Jing Zhang, Chen Wang, Xiaoguo Shi, Qing Feng, Tingting Shen, Siyuan Wang

**Affiliations:** 1 Division of Environmental Science & Engineering, Qilu University of Technology, Shandong Academy of Sciences, Jinan 250353, China; 2 Division of Light Industry, Qilu University of Technology, Shandong Academy of Sciences, Jinan 250353, China

**Keywords:** conjugated polymer, structural regulation, TiO_2_, visible photocatalyst

## Abstract

The photocatalytic activity of titanium dioxide (TiO_2_) is largely hindered by its low photoresponse and quantum efficiency. TiO_2_ modified by conjugated polymers (CPs) is considered a promising approach to enhance the visible light responsiveness of TiO_2_. In this work, in order to investigate the effect of CP structural changes on the photocatalytic performance of TiO_2_ under visible light, trimesoyl chloride–melamine polymers (TMPs) with different structural characteristics were created by varying the parameters of the polymerisation process of tricarbonyl chloride (TMC) and melamine (M). The TMPs were subsequently composited with TiO_2_ to form complex materials (TMP–TiO_2_) using an in situ hydrothermal technique. The photocatalytic activity of TMP–TiO_2_ was evaluated by the degradation of rhodamine B (RhB). The results showed that the trend of the structure of the TMP with the reaction conditions was consistent with the visible light responsiveness of TMP–TiO_2_, and TMP (1:1)–TiO_2_ had the best photocatalytic activity and could degrade 96.1% of the RhB. In conclusion, our study provided new insights into the influence of the structural changes of TMPs on the photocatalytic activity of TMP–TiO_2_ under visible light, and it improves our understanding of how conjugated polymers affect the photocatalytic activity of TiO_2_ under visible light.

## 1. Introduction

The environment and energy are significant influencing variables for the sustainable development of human civilization [1]. The issues of energy and the environment, however, has grown significantly in recent years due to the depletion of conventional fossil fuels such as coal and oil as well as the ongoing degradation of the ecological environment [2]. The use of solar energy has brought new hope to the world [3], and photocatalysis is considered to be one of the most promising green means to solve the problems of energy shortage and environmental pollution due to its advantage of being able to directly use solar energy to drive the reaction [4].

Photocatalysts are at the heart of photocatalytic technology, converting light energy into chemical energy through the excitation of photocatalysts. Among these, titanium dioxide (TiO_2_) is deemed the best candidate due to its high activity and low cost [5]. Unfortunately, its catalytic activity is largely hampered by the low light response and quantum efficiency [6]. Therefore, to address the shortcomings of TiO_2_, various studies have been carried out, such as precious metal deposition, metal ion or nonmetal atom doping, semiconductor compounding, dye sensitization, and conjugated polymer modification [7,8,9,10]. Among them, conjugated polymer modification has attracted more attention from scientists because of its easy preparation and high efficiency and stability.

In recent years, modification of TiO_2_ with conjugated polymers (CPs) has been considered a promising strategy to effectively enhance the visible light absorption and quantum efficiency of TiO_2_ by exploiting the structural properties of the conjugated polymers. Generally, carrier mobility is closely related to the photocatalytic activity of CP/TiO_2_ hybrids, yet the internal structure of CPs is one of the main factors affecting carrier mobility [11]. First, the functional groups of the CPs (e.g., -NH_2_, -NH, and -COOH) can form ideal tight interfacial contacts with TiO_2_, and the tight contacts facilitate effective interfacial charge separation [12]. Second, the large π-conjugated system of CPs provides the condition of carrier delocalization migration [13]. Furthermore, the morphology [14] and electrochemical impedance [15,16] of CPs also affect the carrier migration efficiency, thereby affecting the photocatalytic performance. Thus, based on the above analysis, it is necessary to deeply explore the effect of CP structure on the photocatalytic performance of CP/TiO_2_ hybrid photocatalysts. However, to date, there are relatively few such studies. Notably, the structure and physicochemical properties of CPs are largely affected by polymerization parameters such as reaction temperature, reaction time, and the molar ratio of monomer during the synthetic process of CPs [17,18,19,20,21].

The method for preparing trimesoyl chloride–melamine copolymer (TMP)-modified TiO_2_ was established in our previous study [22], and the visible light responsiveness of TMP-modified TiO_2_ was confirmed. To investigate the effect of the conjugated polymer TMP structure on the visible light responsiveness of the TMP–TiO_2_ complex, different structures, and properties of the conjugated polymer TMPs were successfully prepared by varying the reaction ratio, reaction time, and reaction temperature of tricarbonyl chloride (TMC) and melamine (M), and the different conjugated polymer TMP structures were compounded with TiO_2_ in this paper. The effect of conjugated polymer TMP structural change on the visible light catalytic performance of the subsequent complex TMP–TiO_2_ was investigated. The relationship between the structural properties of TMP and the visible light responsiveness of TMP–TiO_2_ was revealed, and the mechanism of the effect of the conjugated polymer TMP on the visible light catalytic performance of TiO_2_ was better explained.

## 2. Materials and Methods

### 2.1. Chemicals and Reagents

Trimesoyl chloride, and melamine were purchased from Aladdin Chemistry Co., Ltd. (Shanghai, China). Anhydrous copper chloride (CuCl_2_), and tetrabutyl titanate (TBT) were obtained from Shanghai Maclean Biochemical Technology Co., Ltd. (Shanghai, China). Glacial acetic acid (CH_3_COOH) and anhydrous ethanol (EtOH) were provided by Tianjin Fuyu Fine Chemical Co., Ltd. (Tianjin, China). Hydrochloric acid (HCl) was provided by Tianjin Kermel Chemical Reagents Company (Tianjin, China).

### 2.2. Synthesis of TMP Catalyst

**Figure 1:**
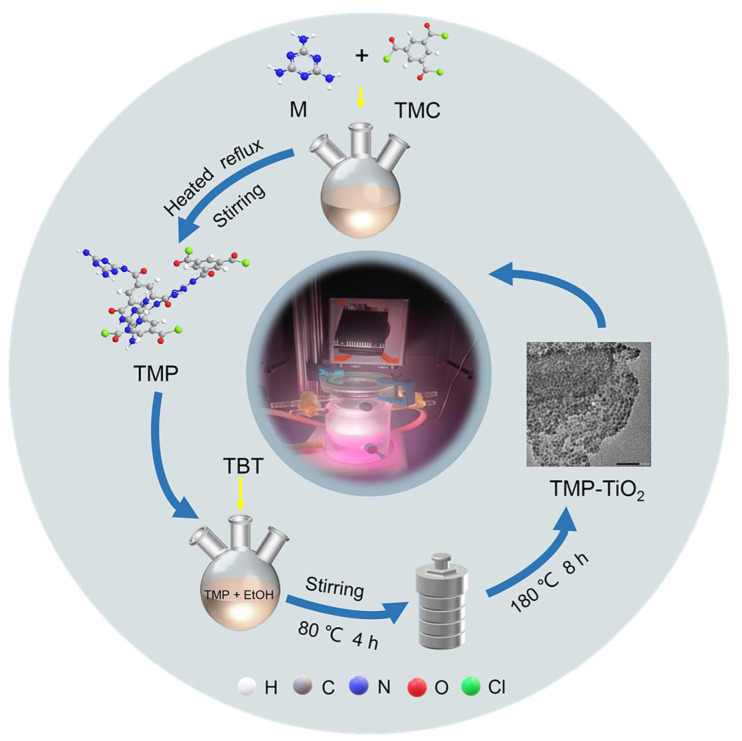
Flow diagram of the material preparation.

The preparation method of TMP was detailed in our previous study [22]. Briefly, a certain amount of trimesoyl chloride was loaded in a 250 mL three-neck flask. Subsequently, melamine was quickly transferred to the flask containing trimesoyl chloride and the catalyst CuCl_2_ (2% of the total material) was then immediately added. Then, the three-necked flask with the reactants was heated in an oil bath and the reactants were stirred. At the end of the reaction, the products were cooled to room temperature and washed repeatedly with deionized water and ethanol to remove impurities. Finally, the TMP was obtained by drying the products at 105 °C for 2 h (Figure 1). During the TMP preparation process, reaction temperature, reaction time, and reactant molar ratio (M: TMC) affect the structure of TMP. Therefore, a series of samples were synthesized by varying the above parameters based on the results of the previous study [22] (Table S1).

### 2.3. Synthesis of TMP–TiO_2_ Composite Catalyst

TMP–TiO_2_ composites were fabricated using a facile in situ hydrothermal method [22]. First, 1 g of synthesized TMP was transferred to a 100 mL volumetric flask containing 100 mL EtOH, and ultrasonically dispersed for 2 h to ensure it was adequately dispersed. Second, the TMP solution was slowly poured into a 300 mL beaker, and 1 mL TBT was added dropwise into it to form a homogeneously mixed solution after constant stirring for 10 min. Then, 1 mL CH_3_COOH and 50 mL distilled water were slowly poured into a 250 mL beaker containing 150 mL EtOH, stirred well, and the pH of the mixture was adjusted with hydrochloric acid to keep the pH at 3. After that, the mixed solution was added dropwise into TMP homogeneous solution in a 500 mL three-necked flask using a separating funnel, which was then stirred and reacted at 80 °C for 4 h in an oil bath. Afterwards, the mixture solution was quickly transferred into a Teflon-lined stainless-steel autoclave, sealed tightly, and kept at 180 °C for 8 h in an oven. After it cooled to room temperature, the products were repeatedly washed with deionized water and ethyl alcohol until the washing solution became colourless. Finally, the TMP–TiO_2_ composites were obtained by drying the samples at 105 °C for 2 h (Figure 1).

### 2.4. Characterization

The analysis of functional group variation of the synthesized catalysts, which was caused by its synthesis parameters changes, was performed using Fourier transform infrared spectroscopy (FT-IR) using KBr as background on a Shimadzu IRAffinity-1S FT-IR spectrometer in the wavelength range from 400 to 4000 cm^−1^ at room temperature. The dried catalysts (0.01 mg) and KBr (10 mg) powders were accurately weighed using analytical scales (accuracy: ±0.0001 mg) and were combined to form mixtures in an agate mortar with infrared lamp irradiation. Once completely mixed (after grinding for about 5 min), the obtained mixtures were formed into a circular pellet with a tableting tool for 10 min at 0.5 MPa. Then, the pellet was quickly placed into the FT-IR system for IR spectroscopy; before measuring the mixed sample, the KBr background had been subtracted. After the measurement, infrared spectrum analysis was performed with Origin Pro (OriginLab, Northampton, MA, USA).

The morphology was determined by transmission electron microscopy (TEM).

The electronic energy band gap (Eg) values of the prepared catalysts were obtained by ultraviolet–visible diffuse reflectance spectroscopy (UV-Vis DRS) obtained using a UV/vis/NIR spectrophotometer (UV-3600Plus, Shimadzu, Kyoto, Japan) equipped with an integrating sphere.

The electrochemical impedance spectroscopy (EIS), cyclic voltammetry (CV), and photocurrent response were obtained using a CHI-660E electrochemical analyser (CH Instruments, Shanghai, China). The working electrode, counter electrode, and reference electrode were an ITO electrode, platinum electrode, and saturated Ag/AgCl electrode, respectively. A Bu_4_NPF_6_ solution (0.1 mol/L) was used as the electrolyte, and ferrocene was used as the internal standard. The scan rate was 50 mV/s. Under these experimental conditions, the potential value of ferrocene was measured to be 0.29 eV, while the potential value of ferrocene in a vacuum was 4.8 eV. Therefore, the calibration parameter can be obtained as 4.51 eV. The highest occupied molecular orbital (HOMO) energy level of the TMP polymer can be calculated from the corresponding equation HOMO = −e (φ_ox_ + 4.8 − φ_Fe/Fe+_) (eV) for the starting oxidation potential of the polymer, and the lowest unoccupied molecular orbital (LUMO) energy level can be obtained by combining the Eg obtained from the UV–Vis DRS diffuse reflectance spectroscopy [23,24].

The chemical composition and valance state measurements were performed by X-ray photoelectron spectroscopy (XPS, Thermo Scientific K-Alpha, Waltham, MA, USA).

### 2.5. Measurement of Photocatalytic Activity

The photocatalytic activity of samples was tested by photocatalytic degradation of rhodamine B (RhB) in water. A total of 150 mL of a RhB (20 mg/L) solution was measured and added to the reactor; then, 0.15 g of TMP–TiO_2_ was weighed and added to the reactor, and a suspension was obtained by thorough stirring. Before starting the photocatalytic reaction, the suspension was placed under dark conditions and stirred magnetically to reach the adsorption–desorption equilibrium. The suspension was placed under a 300 W xenon light source at wavelengths greater than 400 nm for 1.5 h with continuous stirring to bring the TMP–TiO_2_ into uniform contact with the RhB molecules. During the light period, the condensate was passed around the reactor to ensure that the reaction temperature of the mixture in the system remained constant. The solution was taken from the mixture at each time interval, filtered through a needle filter (0.22 μm), and the absorbance value was measured by a UV spectrophotometer to measure the absorbance after degradation and calculate the degradation rate of RhB (Figure 1).

## 3. Results

### 3.1. Analysis of Structural Changes in TMP

#### 3.1.1. FT-IR Profiling

**Figure 2:**
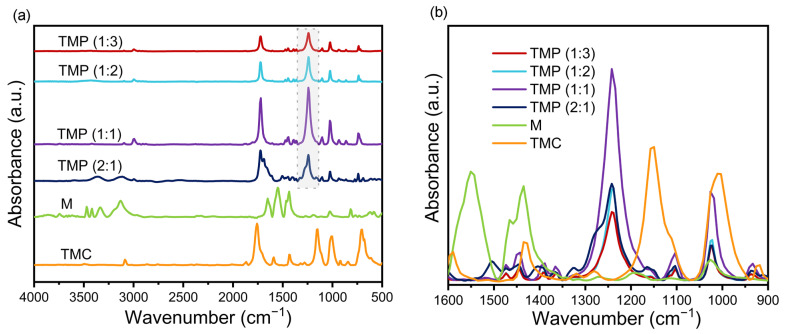
(**a**) Fourier transform infrared spectroscopy (FT-IR) of melamine (M), tricarbonyl chloride (TMC), and trimesoyl chloride–melamine copolymer (TMP) after baseline correction (500–4000 cm^−1^); (**b**) FT-IR spectra of M, TMC, and TMP after baseline correction (900–1600 cm^−1^).

As shown in Figure 2a,b, the new absorption band at 1242 cm^−1^ in the TMP was attributed to the C-N stretching vibration of the secondary amide III band [25], which was not observed in melamine and trimesoyl chloride. This result demonstrated that the TMP polymer was successfully prepared by forming the C-N bond via the poly-condensation reaction of melamine with tricarbonyl chloride.

The greater the absorption strength of the C-N bond, the more complete the reaction tends to be, the greater the π-conjugated effect [26], and the greater the ability of the TMP to transfer carriers. As shown in Figure 2b, the intensity of the C-N stretching vibration in the conjugated polymer TMP was markedly affected by the ratio of melamine to trimesoyl chloride. The absorption strength of the C-N bond roughly tended to increase and then decrease as the proportion of trimesoyl chloride increased. As the amount of melamine in the system remained constant, the more trimesoyl chloride was added, the more complete the reaction would be. When the amount of trimesoyl chloride added was over the amount required for the melamine reaction, any further trimesoyl chloride would only react with the resulting TMP and prevent it from polymerizing into a longer chain polymer [27,28].

As shown in Figure S1a,b, the intensity of the C-N stretching vibration in the conjugated polymer TMP was affected by the polycondensation reaction temperature of the melamine and trimesoyl chloride. With the increase in the polycondensation reaction temperature, the C-N bond absorption intensity showed a trend of increasing and then decreasing. With the change in polymerization reaction temperature, the possibility of collision between melamine and trimesoyl chloride and the mode of polymerization would also change [29]. At lower polycondensation reaction temperatures, the number of active sites increases as the reaction temperature increases [30], and the melamine and trimesoyl chloride form TMP polymers, resulting in the formation of more C-N bonds. As the reaction temperature of melamine and trimesoyl chloride continued to rise, the viscosity of the condensation to produce TMP increased, causing the thermal movement of the unreacted melamine and trimesoyl chloride molecules in the reaction system to be bound and the condensation reaction of both to be hindered; therefore, the C-N bond was reduced accordingly.

As shown in Figure S1c,d, the C-N bond absorption intensity tended to increase and then decrease as the reaction time increased between melamine and trimesoyl chloride. The extent of the polymerization reaction proceeded with increased reaction time between melamine and trimesoyl chloride, with an increase in the monomer conversion rate, resulting in more C-N bonds being formed and the reaction tending from incomplete to complete. Once the time limit for maximum conversion of the melamine and trimesoyl chloride monomer is reached, a further increase in reaction time leads to an increase in side reactions such as chain exchange and chain transfer, destroying the resulting polymer TMP and causing a reduction in the C-N bond peak [31].

#### 3.1.2. TEM Images

**Figure 3:**
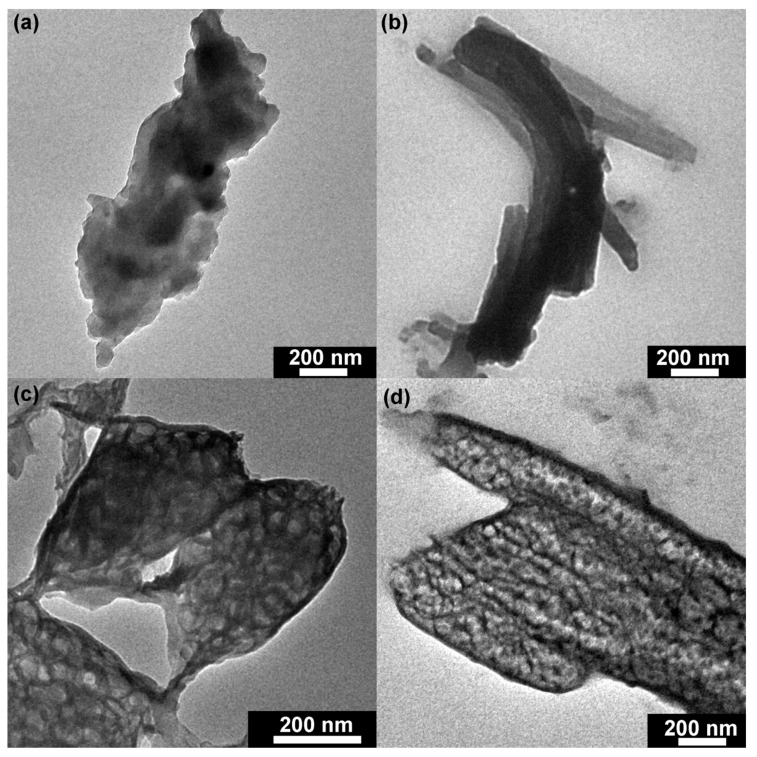
Transmission electron microscopy (TEM) of TMP synthesized by different M:TMC ratios (**a**) M:TMC= 1:3; (**b**) M:TMC= 1:2; (**c**) M:TMC= 1:1; (**d**) M:TMC= 2:1.

TEM techniques were executed to further investigate the structure of the synthesized catalysts. As shown in Figure 3a, TMP (1:3) displayed an irregular structure, which was formed by the agglomeration of small particles. With increased molar ratio, the structure of TMP (1:2) showed a lamellar stack structure, which indicated that the molar ratio could significantly affect the structure of TMP polymers (Figure 3b). Figure 3c illustrates that TMP (1:1) was a porous sheet structure. Compared with TMP (1:1), the structure of TMP (2:1) was a mixture of porous rods and sheets (Figure 3d). Based on the above results, it was reasonable to propose that the variation of the molar ratio between M and TMC could cause the structure change of TMP polymers [32].

#### 3.1.3. Band Gap Analysis

**Figure 4:**
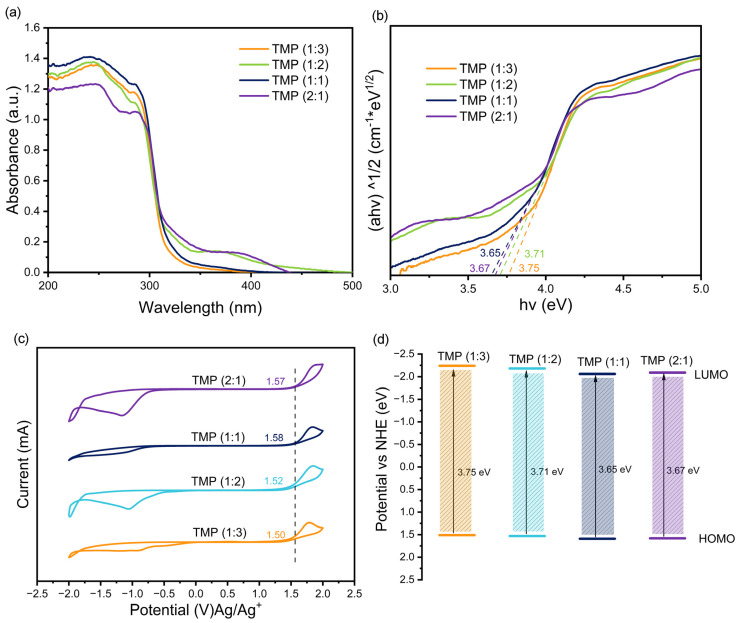
(**a**) Ultraviolet–visible diffuse reflectance spectroscopy (UV-Vis DRS) of the TMP. (**b**) Diagram of the Kubelka–Munk function of the TMP versus the absorbed light energy (band gap width). (**c**) Cyclic voltammogram (CV) of TMP. (**d**) Location of TMP highest occupied molecular orbital (HOMO) and lowest unoccupied molecular orbital (LUMO).

**Figure 5:**
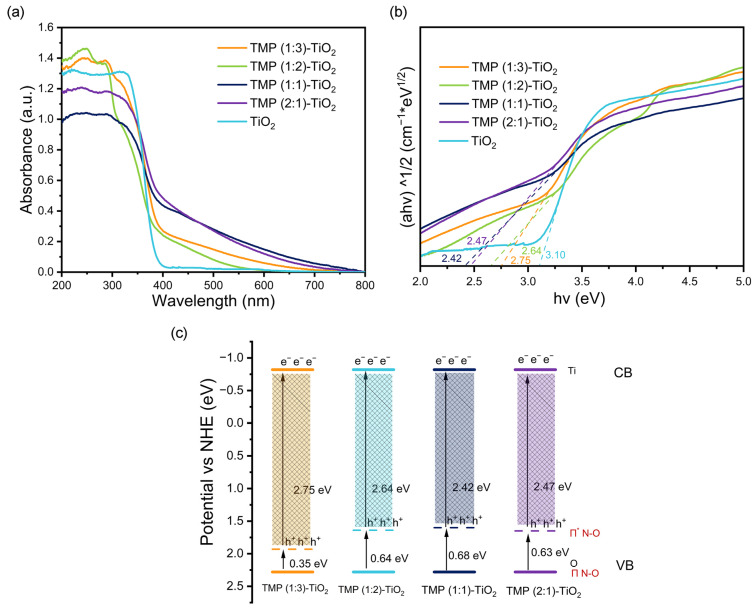
(**a**) UV-Vis DRS of the TMP–TiO_2_**.** (**b**) Diagram of the Kubelka–Munk function of the TMP–TiO_2_ versus the absorbed light energy (band gap width). (**c**) Location of TMP–TiO_2_ conduction band (CB) and valence band (VB).

The Kubelka–Munk function and Tauc relationship were applied to distinguish the Eg of the materials [33]. According to Figure 4a,b, the HOMO–LUMO band gaps of TMP (1:3), TMP (1:2), TMP (1:1), and TMP (2:1) were obtained as 3.75 eV, 3. 71 eV, 3.65 eV, and 3.67 eV, respectively [34]. They were in good agreement with the trends of band gap widths (2.75 eV, 2.64 eV, 2.42 eV, 2.47 eV) of the composites: TMP (1:3)–TiO_2_, TMP (1:2)–TiO_2_, TMP (1:1)–TiO_2_, and TMP (2:1)–TiO_2_, respectively (Figure 5a,b). The oxidation potentials φ_ox_ of TMP (1:3), TMP (1:2), TMP (1:1), and TMP (2:1) were 1.50 eV, 1.52 eV, 1.58 eV, and 1.57 eV, respectively, as shown in Figure 4c and determined by cyclic voltammetry [35]. The energy levels of the HOMO were 1.51 eV, 1.53 eV, 1.59 eV, and 1.58 eV (vs. NHE), calculated from the equations in 2.4. The LUMOs of TMP (1:3), TMP (1:2), TMP (1:1), and TMP (2:1) were calculated to be −2.24 eV, −2.18 eV, −2.06 eV, and −2.09 eV, respectively, based on the HOMO and band gap of the TMP polymer [36], as shown in Figure 4d. As the ratio of the TMP synthesis reactant melamine to trimesoyl chloride increased, the LUMO position of the product TMP shifted down and then up [37,38], in line with the trend of the forbidden band of the TMP–TiO_2_ complex in Figure 5c (TiO_2_ valence band top rise).

As can be seen from Figure S2a,b, by varying the reaction temperature of melamine and trimesoyl chloride (from 75 °C to 95 °C), the HOMO–LUMO band gaps of the synthesized TMP were 3.78 eV, 3.76 eV, 3.65 eV, and 3.80 eV respectively. They were in good agreement with the trends of band gap widths (2.82 eV, 2.79 eV, 2.42 eV, 2.85 eV) of the composites [39]: TMP (75 °C)–TiO_2_, TMP (85 °C)–TiO_2_, TMP (95 °C)–TiO_2_, and TMP (105 °C)–TiO_2_ (Figure S4a,b). TMP (75 °C), TMP (85 °C), TMP (95 °C), and TMP (105 °C) had oxidation potentials φ_ox_ of 1.53 eV, 1.52 eV, 1.58 eV, and 1.53 eV, respectively, as determined by cyclic voltammetry [40], and is shown in Figure S2c. Calculated from the equations, the energy levels of the HOMO were 1.54 eV, 1.53 eV, 1.59 eV, and 1.54 eV (vs. NHE), respectively [41]. The LUMOs of TMP (75 °C), TMP (85 °C), TMP (95 °C), and TMP (105 °C) were calculated to be −2.24 eV, −2.23 eV, −2.06 eV, and −2.26 eV, respectively, based on the HOMO and band gap width of the TMP polymer as shown in Figure S2d [42]. As the reaction temperature of melamine and trimesoyl chloride increased, the LUMO position of the resulting TMP first decreased and then shifted upwards, in line with the trend of the forbidden band of the TMP–TiO_2_ complex in Figure S4e.

As shown in Figure S3a,b, the band gap of the synthesized TMP increased and then decreased with increasing reaction time of melamine and trimellitic chloride, which was consistent with the trend of band gap change of TMP–TiO_2_ in Figure S4c,d. The oxidation potentials φ_ox_ of TMP (3 h), TMP (4 h), TMP (5 h), and TMP (6 h) were 1.59 eV, 1.58 eV, 1.62 eV, and 1.63 eV, respectively, as shown in Figure S3c and determined by cyclic voltammetry [43]. Calculated from the equations, the HOMO energy levels were 1.60 eV, 1.59 eV, 1.63 eV, and 1.64 eV (vs. NHE), respectively. The LUMOs of TMP (3 h), TMP (4 h), TMP (5 h), and TMP (6 h) were calculated to be −2.16 eV, −2.06 eV, −2.07 eV, and −2.08 eV, respectively, based on the HOMO and band gap of the TMP polymer, as shown in Figure S3d. As the reaction time between melamine and trimesoyl chloride increased, the HOMO position of the resulting TMP moved up and then down, in line with the trend of the forbidden band of the TMP–TiO_2_ complex in Figure S4f.

The reaction conditions for the synthesis of TMP by the reaction of melamine with trimesoyl chloride affected the formation of C-N bonds and therefore the π-conjugated effect of TMP, which in turn affected the band gap width of TMP and the position of the HOMO and LUMO [44,45]. The additional stabilization energy gained by the TMP system was the off-domain energy because the conjugation effect caused the electron activity range to expand [46,47]. The greater the degree of conjugation, the higher the degree of delocalization, the smaller the energy difference between the HOMO and LUMO, and the smaller the band gap [48].

#### 3.1.4. EIS Analysis

**Figure 6:**
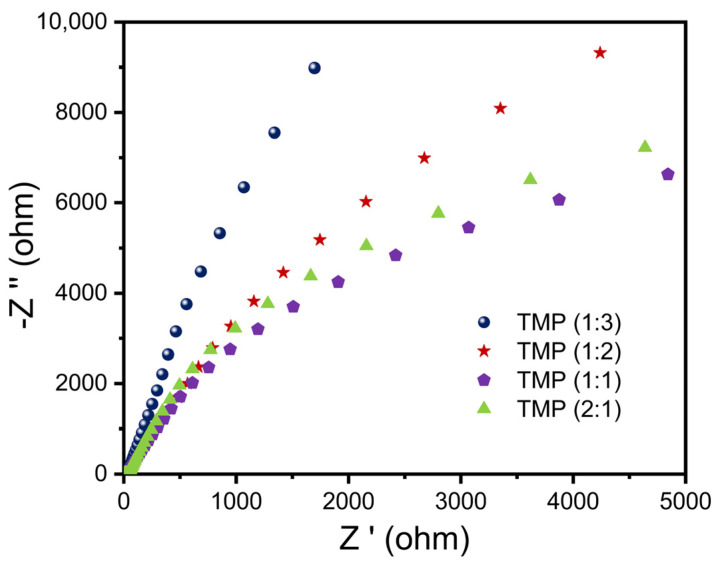
Electrochemical impedance spectroscopy (EIS) of TMP.

In order to explore the influence of parameter changes on the photogenerated charge carrier transfer ability of the TMP polymer during synthesis, an electrochemical impedance test was conducted, and specific analysis was also carried out through the Nyquist atlas. As shown in Figure 6, the EIS of TMP decreased and then increased as the proportion of trimesoyl chloride increased, so the ability of TMP to transfer electrons and holes increased and then decreased as the proportion of trimesoyl chloride increased [49,50,51]. In Figure S5a, the EIS of the resulting TMP decreased and then increased as the temperature of the reaction between melamine and trimesoyl chloride increased, thus the ability of TMP to transfer electrons and holes increased and then decreased as the temperature of the reaction between melamine and trimesoyl chloride increased. The EIS of the resulting TMP decreased and then increased as the reaction time between the melamine and trimesoyl chloride increased, as shown in Figure S5b. Therefore, the ability of TMP to transfer electrons and holes increased and then decreased as the reaction time between melamine and trimesoyl trichloride increased.

### 3.2. Analysis of the Effect of Structural Changes of TMP on the Catalytic Performance of TMP–TiO_2_ under Visible Light

The photocatalytic activity of the synthesized catalysts was evaluated by degrading RhB, which is a typical organic pollutant presented in water, under visible light irradiation. The widely accepted Langmuir–Hinshelwood (L–H) kinetic model was used to describe the photocatalytic kinetics during the photodegradation of RhB in solution, which can be expressed by the following equation [52,53]:

r = −dC/dt = kKC/(1 + KC)(1)

where r represents the reaction rate (mg/L min), k is the reaction rate constant, K is the Langmuir equilibrium adsorption constant of the reactant (L/mg), and C represents the concentration of pollutants in the solution (mg/L). Generally, when the concentration of pollutants in the solution is > 5 mM, the values of K >> 1. Thus, Equation (1) can be rewritten to apparent zero-order Equation (2)

r = −dC/dt = k(2)

However, when the concentration of pollutants in the solution is <1 mM, the values of K << 1. Thus, a classical first-order Equation (3) can be obtained:

r = −dC/dt = kKC(3)

The term kK can be expressed as an apparent rate constant (k_app_; min^−1^) according to Equation (4).

k_app_ = kK (4)

Therefore, Equation (3) can be rewritten as Equation (5)

r = −dC/dt = k_app_ C (5)

Integrating Equation (5) yields Equation (6).

ln (C_t_/C_0_) = −k_app_ t (6)

where C_t_ and C_0_ are the concentration of RhB in solution at time t and 0, respectively.

In addition, when C_t_ = 1/2 C_0_, Equation (6) can be reduced to Equation (7).

t_1/2_ = ln2/k_app_ (7)

**Figure 7:**
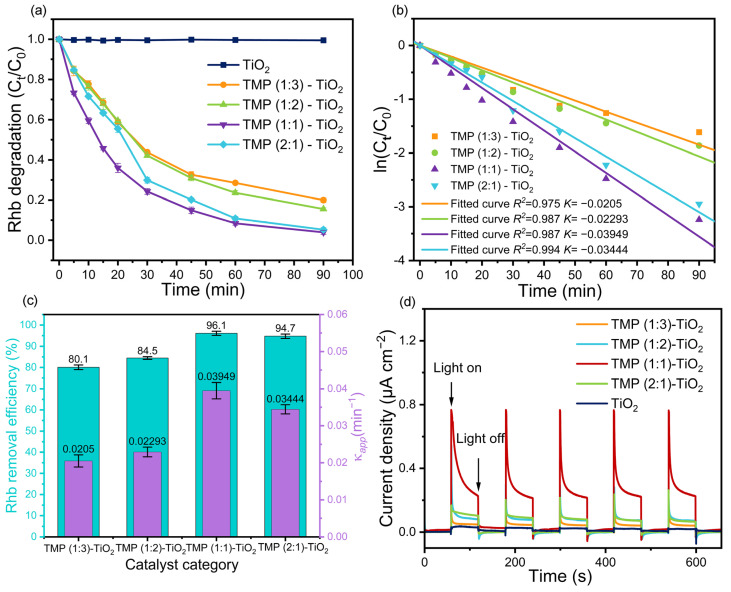
(**a**) Degradation of RhB. (**b**) Hinshelwood plot for studying the kinetic of the process. (**c**) Removal rate of RhB and value of the linear fit κ*_app_*. (**d**) Photocurrent response diagram.

The photocurrent response and RhB degradation experimental results of the various prepared compound catalysts are displayed in Figure 7, Figures S6 and S7, respectively.

In the adsorption process, the RhB removal efficiencies of TMP (1:3)–TiO_2_, TMP (1:2)–TiO_2_, TMP (1:1)–TiO_2_, and TMP (2:1)–TiO_2_ were 18.7%, 20.8%, 36.6%, and 27.9%, respectively (Figure S8a). As shown in Figure 7a, the RhB was only slightly degraded by pure TiO_2_ under visible light, which may be due to the poor visible light absorption performance of TiO_2_ derived from its large gap band [54]. Obviously, compared to pure TiO_2_, the RhB degradation rates of the composite catalysts (TMP (1:3)–TiO_2_, TMP (1:2)–TiO_2_, TMP (1:1)–TiO_2_, TMP (2:1)–TiO_2_) were markedly increased, indicating the composite catalysts have excellent photocatalytic activity. As shown in Figure 7b, the photocatalytic degradation process of RhB was well fitted to Hinshelwood law (Equation (6)). According to Equation (7), the RhB degradation half times (t_1/2_) of TMP (1:3)–TiO_2_, TMP (1:2)–TiO_2_, TMP (1:1)–TiO_2_, TMP (2:1)–TiO_2_ were 33.80 min, 30.22 min, 17.55 min, and 20.12 min, respectively [55,56,57]. Figure 7c illustrates that the reaction rate constant (κ*_app_*) and RhB removal efficiency were noticeably affected by changes in monomer ratio, in which TMP (1:1)–TiO_2_ had the highest κ*_app_* values (0.03949 min^−1^) and RhB removal efficiency (96.1%). According to photocurrent response curves of the synthesized catalysts (Figure 7d), we can suggest that a suitable monomer ratio was not only conducive to improving the light current response but can also remarkedly improved photocurrent density, which was facilitated by rapid separation of photo-generated holes and electrons, leading to enhanced catalytic activity.

In the adsorption process, the RhB removal efficiencies of TMP (75 °C)–TiO_2_, TMP (85 °C)–TiO_2_, TMP (95 °C)–TiO_2_, and TMP (105 °C)–TiO_2_ were 30.1%, 31.6%, 36.6%, and 27.9%, respectively (Figure S8b). The RhB degradation rates of pure TiO_2_ and the composite catalysts (TMP (75 °C)–TiO_2_, TMP (85 °C)–TiO_2_, TMP (95 °C)–TiO_2_, TMP (105 °C)–TiO_2_) are shown in Figure S6a, and indicate that the composite catalysts had stronger RhB degradation rates than pure TiO_2_. As shown in Figure S6b, the photocatalytic degradation process of RhB was well fitted to Hinshelwood law (Equation (6)). By Equation (7), the RhB degradation half times (t_1/2_) of TMP (75 °C)–TiO_2_, TMP (85 °C)–TiO_2_, TMP (95 °C)–TiO_2_, TMP (105 °C)–TiO_2_ were 19.67 min, 18.82 min, 17.55 min, and 29.04 min, respectively. As shown in Figure S6c, with increased synthesis temperature of TMP, the final RhB removal efficiency of the composite catalyst showed little change after 90 min, but the κ*_app_* values showed clear differences, which gradually increased from 0.03523 min^−1^ to 0.03949 min^−1^ and then decreased to 0.03444 min^−1^. This changing trend corresponded with the variation of the photocurrent response of TMP (75 °C)–TiO_2_, TMP (85 °C)–TiO_2_, TMP (95 °C)–TiO_2_, and TMP (105 °C)–TiO_2_ (Figure S6d). Thus, based on this, we can conclude that the increased synthesis temperature of TMP can influence its light current response and photocurrent density. The enhanced photocurrent density was beneficial to the generated reactive oxygen radicals due to the rapid separation of photo-generated holes and electrons, resulting in the highest κ*_app_* values. The confined catalytic ability of the composite catalysts at certain experiment conditions were similar to the final RhB removal efficiency of the various composite catalysts.

In the adsorption process, the RhB removal efficiencies of TMP (3 h)–TiO_2_, TMP (4 h)–TiO_2_, TMP (5 h)–TiO_2_, and TMP (6 h)–TiO_2_ were 22.4%, 36.6%, 26.6%, and 22.5%, respectively (Figure S8c). Figure S7a indicated there was obvious differences in the RhB degradation rate between pure TiO_2_ and composite catalysts (TMP (3 h)–TiO_2_, TMP (4 h)–TiO_2_, TMP (5 h)–TiO_2_, TMP (6 h)–TiO_2_), in which the composite catalyst showed superior catalytic activity, confirmed by the well fitted curves of the Langmuir–Hinshelwood (L–H) kinetic model (Figure S7b). In addition, according to Equation (7), the RhB degradation half times (t_1/2_) of TMP (3 h)–TiO_2_, TMP (4 h)–TiO_2_, TMP (5 h)–TiO_2_, and TMP (6 h)–TiO_2_ were 28.94 min, 17.55 min, 21.31 min, and 27.38 min, respectively. As shown in Figure S7c, with the synthesized time of TMP increased from 3 h to 4 h, the κ*_app_* values and RhB removal efficiency of TMP (3 h)–TiO_2_ and TMP (4 h)–TiO_2_ markedly increased, and then gradually decreased from 4 h to 6 h. A similar trend was seen in the light current response and photocurrent density (Figure S7d). Generally, the higher the light current response and photocurrent density of the photocatalyst the more easily it is excited by light with faster migration of photo-generated electrons to prevent their recombination with photo-generated holes [55], resulting in a higher RhB removal efficiency. Based on the above results, we can conclude that the synthesis time of the TMP significantly impacted TMP–TiO_2_ photocatalytic performance, which may be due to the different synthesis times causing changes to the TMP’s own structure and then impact the TMP–TiO_2_ light current response and photocurrent density.

Moreover, in order to further evaluate the photocatalytic performance of the synthesized catalysts in a rational way, the comparation of this work with reported references was implemented. As shown in Table S2, the TMP (1:1)–TiO_2_ obtained the highest RhB removal efficiency, indicating that TMP (1:1)–TiO_2_ had excellent photocatalytic activity. In addition, TMP (1:1)–TiO_2_ had a higher RhB removal efficiency over a shorter irradiation time even with a higher concentration of RhB. Thus, the TMP (1:1)–TiO_2_ is a promising photocatalyst.

### 3.3. Photocatalytic Mechanism of TMP–TiO_2_ under Visible Light

**Figure 8:**
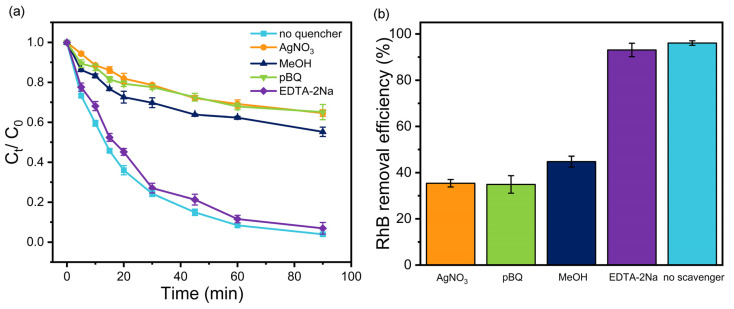
Effects of scavengers on the degradation of RhB. (**a**) Photocatalytic removal curves of RhB. (**b**) Photocatalytic removal efficiency of RhB.

Quenching experiments were implemented to investigate the active components and reaction mechanism of RhB degradation by TMP (1:1)–TiO_2_ during the photocatalytic process. AgNO_3_, pBQ, MeOH, and EDTA-2Na were used as quenchers of electrons (e^−^), superoxide radicals (O_2_^−^), hydroxyl radicals (·OH) and holes (h^+^), respectively. As shown in Figure 8, the RhB removal efficiency was markedly affected after the addition of scavengers. The inhibitory effect of EDTA-2Na was slight, indicating that the contribution of h^+^ is negligible. Generally, during the photocatalytic process, the O_2_^−^ could be generated according to Equation (8) [56]:

e^−^ + O_2_ → O_2_^−^ (8)

Thus, the similar inhibiting effect of e^−^ and O_2_^−^ indicated that O_2_^−^ plays an important role in RhB degradation. However, pBQ could not only rapidly react with O_2_^−^ (8.3 × 10^8^ M^−1^s^−1^) but also with ·OH (1.2 × 10^9^ M^−1^s^−1^) [57]. The ·OH could be generated according to Equations (9)–(11) [58,59]:

h^+^ + OH^−^ → ·OH (9)

h^+^ + H_2_O → ·OH + H^+^ (10)

O_2_^−^ + H_2_O → ·OH + OH^−^ (11)

Nevertheless, the ·OH generation by TMP (1:1)–TiO_2_ could not be obtained through Equations (2) and (3), due to its VB edge potential being more negative than the reduction potential of OH^−^/·OH (1.99 eV) and H_2_O/·OH (2.3 eV) [60]. The RhB degradation was also inhibited by MeOH, but the inhibitory effect was weaker than that with pBQ. Notably, MeOH does not reacted with O_2_^−^ but could rapidly react with ·OH (9.7 × 10^8^ M^−1^s^−1^), similar to pBQ [61]. Thus, it is reasonable to propose that ·OH was the dominant oxidant for RhB degradation. The different inhibitory effects between pBQ and MeOH suggested that O_2_^−^ is involved in the generation of ·OH.

**Figure 9:**
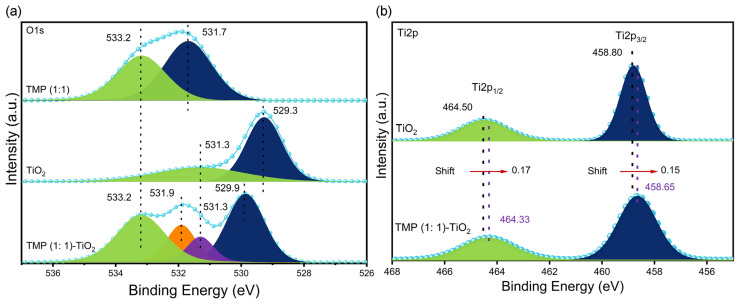
(**a**) XPS O1s spectra of TMP (1:1), TiO_2_, and TMP (1:1)–TiO_2_; (**b**) XPS Ti2p spectra of TMP (1:1), TiO_2_, and TMP (1:1)–TiO_2_.

As shown in Figure 9a, the O1s spectrum of TMP (1:1) has two fitting peaks. The peak at binding energy 531.7 eV is due to the C-O bond formed by hydrolysis of the acyl chloride bond, and the peak at binding energy 533.2 eV is a C=O bond. The O1s spectrum of TiO_2_ has two fitting peaks: the peak at the binding energy of 529.3 eV is a Ti-O-Ti bond, and the peak at the binding energy of 531.3 eV is the Ti-OH bond formed by the hydroxyl oxygen of the surface hydroxyl group in TiO_2_. By comparison, the fitting peaks of 529.9 eV, 531.3 eV, 531.9 eV, and 533.2 eV in the TMP (1:1)–TiO_2_ complex correspond to the Ti-O-Ti bond, Ti-O-N, C-O bond, and C=O bond in TiO_2_, respectively [62,63]. Moreover, the binding energy was relatively offset, indicating that TMP (1:1) reacted with TiO_2_, generating heterogeneous energy levels in TiO_2_, so the band gap width of TiO_2_ was changed.

As shown in Figure 9b, Ti2p3/2 and Ti2p1/2 are the characteristic peaks of TiO_2_ at the binding energy of 458.8 eV and 464.5 eV [64,65]. In the TMP (1:1)–TiO_2_ complex, the characteristic peaks of TiO_2_ were all shifted (redshifted) towards low binding energies by 0.15 eV and 0.17 eV, respectively. The redshift phenomenon can be attributed to the formation of Ti-O-N bonds in the complex. Since the electronegativity of O is greater than that of N, the density of the electron cloud around the Ti^4+^ ions increased, thus placing Ti^4+^ ions in a chemical environment where electrons are available [66,67], resulting in a shift towards lower binding energies (redshift).

**Figure 10:**
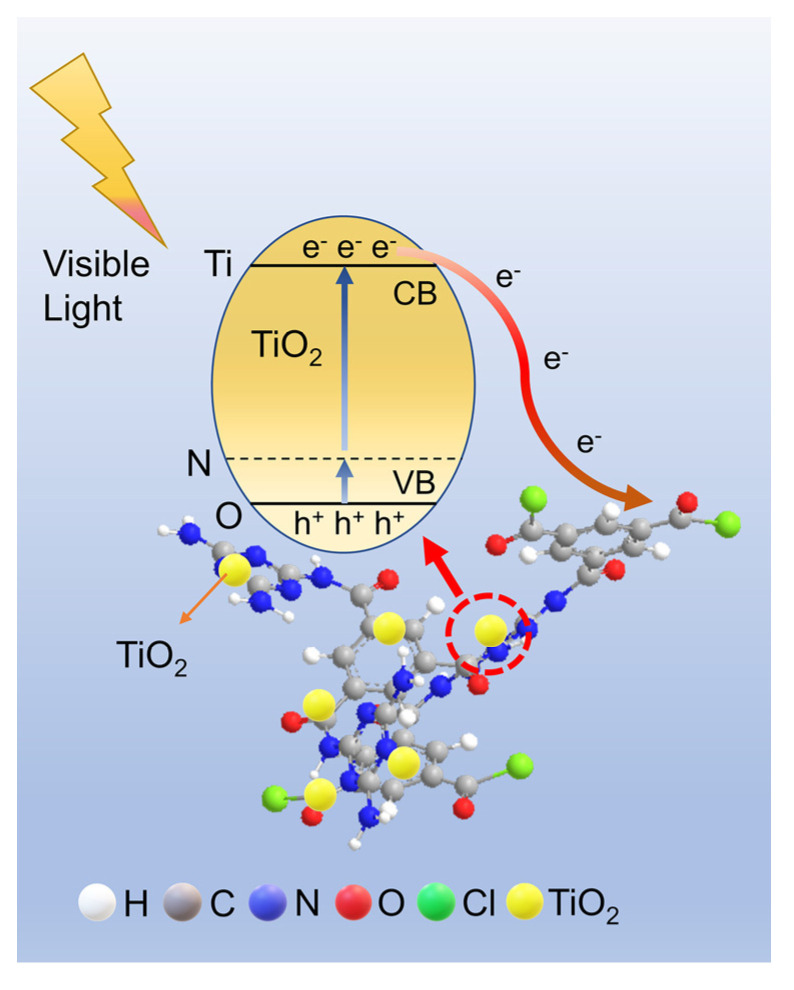
Photocatalytic mechanism of photo-generated carrier separation and transfer on TMP–TiO_2_ sample under visible light.

As shown in Figure 10, TMP is compounded with TiO_2_ to form Ti-O-N bond, resulting in the appearance of stray energy levels in the TiO_2_ band gap and decrease of the band gap width. Moreover, the TMP π-conjugated system is a channel for electron transport, which accelerates electron transfer and improves electron and hole separation efficiency, resulting in TMP–TiO_2_ having good photocatalytic performance in visible light.

## 4. Conclusions

Based on the FT-IR, UV-vis DRS, TEM, EIS, CV, and XPS results, and the degradation rate of RhB, we concluded that the different structures of TMP were produced by varying the reaction ratios, reaction temperatures, and reaction times of the reactants melamine and trimesoyl chloride. The bonding profile, π-conjugated effect, morphology, and electrochemical impedance of the resulting TMP were changed. As the proportion of trimesoyl chloride increased, the strength of the resulting amide bond strengthened and then weakened, the π-conjugated effect of the TMP increased and then decreased, the electrochemical impedance decreased and then increased, and the ability of the TMP to transfer photogenerated electrons and holes increased and then decreased. After compounding TMP with TiO_2_, the structure of TMP further influenced the visible light responsiveness and photocatalytic performance of the TMP–TiO_2_ complex, and the visible light responsiveness and photocatalytic performance of TMP–TiO_2_ showed an increased and then weakened trend. Similar conclusions were reached by changing other parameter conditions.

## Data Availability

Not applicable.

## Supplementary Materials

The following supporting information can be downloaded at: https://www.mdpi.com/article/10.3390/ma16041563/s1. Refs. [68,69,70,71,72,73] are cited in the supplementary materials.
