# Supplementary material for: Modulation of the Structure of the Conjugated Polymer TMP and the Effect of Its Structure on the Catalytic Performance of TMP–TiO2 under Visible Light: Catalyst Preparation, Performance and Mechanism
Source: Materials (Basel). 2023 Feb 13;16(4):1563. doi: 10.3390/ma16041563 (PMC9965725; doi:10.3390/ma16041563)
Supplement: Supplementary file 1 [file materials-16-01563-s001.zip › materials-2187612-supplementary.pdf]

materials

Supplementary for Article

Modulation of the Structure of the Conjugated Polymer TMP and the Effect of Its Structure on the Catalytic Performance of TMP–TiO_2 under Visible Light: Catalyst Preparation, Performance and Mechanism

Jing Zhang [1], Chen Wang [1,*], Xiaoguo Shi [1], Qing Feng [1], Tingting Shen [1] and Siyuan Wang [2]

[1] Division of Environmental Science & Engineering, Qilu University of Technology, Shandong Academy of Sciences, Jinan 250353, China
[2] Division of Light Industry, Qilu University of Technology, Shandong Academy of Sciences, Jinan 250353, China
* Correspondence: shanqing123@qlu.edu.cn

Table S1. Experimental schemes.

Number	Reaction temperature (℃)	Reaction time (h)	Reactant molar ratios (M: TMC)	Sample code
1	75	4	1: 1	TMP (75 °C)
2	85	4	1: 1	TMP (85 °C)
3	95	4	1: 1	TMP (95 °C)
4	105	4	1: 1	TMP (105 °C)
5	95	3	1: 1	TMP (3 h)
6	95	4	1: 1	TMP (4 h)
7	95	5	1: 1	TMP (5 h)
8	95	6	1: 1	TMP (6 h)
9	95	4	1: 3	TMP (1: 3)
10	95	4	1: 2	TMP (1: 2)
11	95	4	1: 1	TMP (1: 1)
12	95	4	2: 1	TMP (2: 1)

* Trimesoyl chloride-melamine copolymer (TMP), melamine (M) and tricarbonyl chloride (TMC).
* The temperature given is the temperature of the external oil bath.

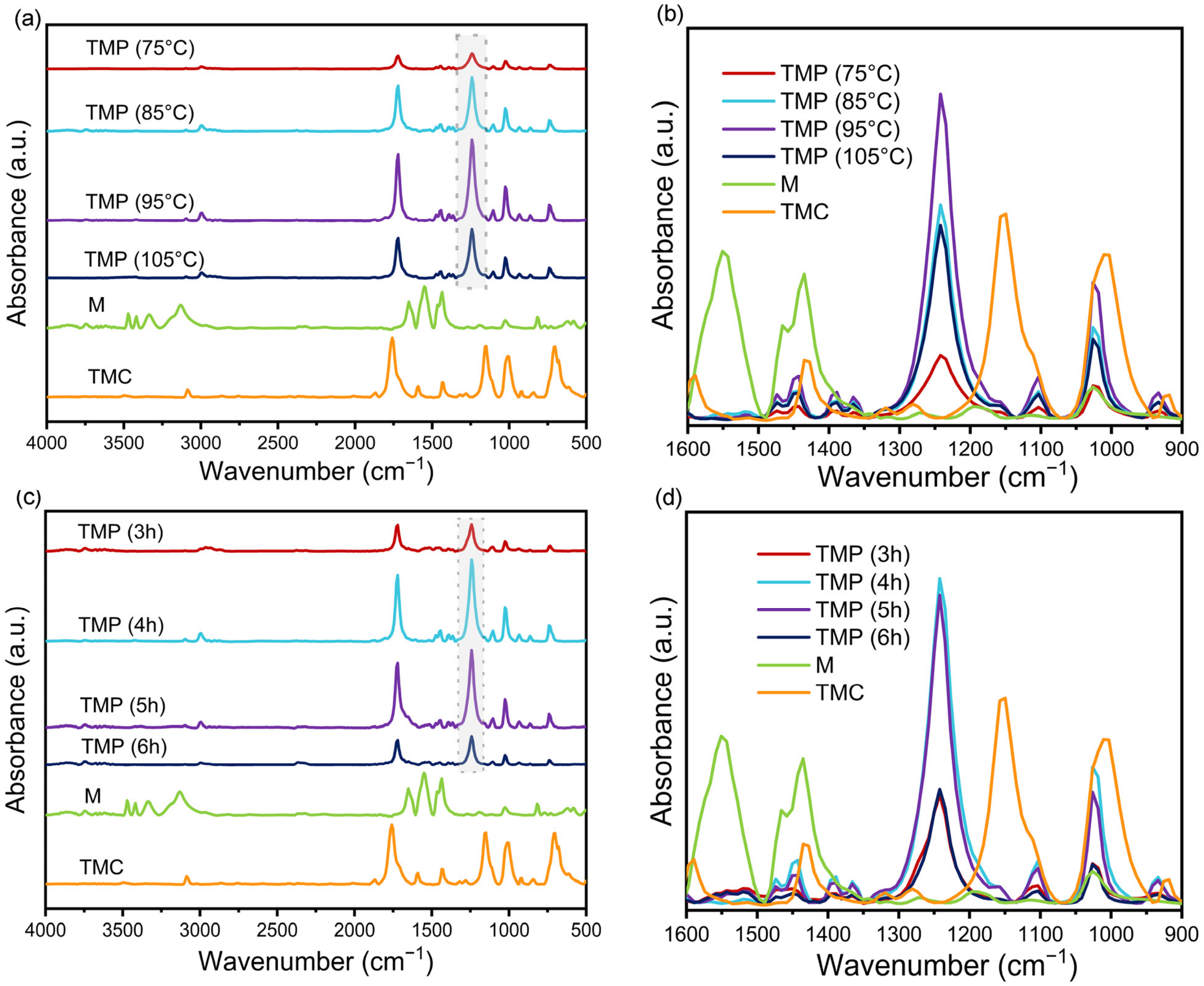

Figure S1. FT-IR spectra of TMP: (**a**) Different reaction temperatures of M and TMC (500-4000 cm^{-1}); (**b**) Different reaction temperatures of M and TMC (900-1600 cm^{-1}); (**c**) Different reaction time of M and TMC (500-4000 cm^{-1}); (**d**) Different reaction time of M and TMC (900-1600 cm^{-1}).

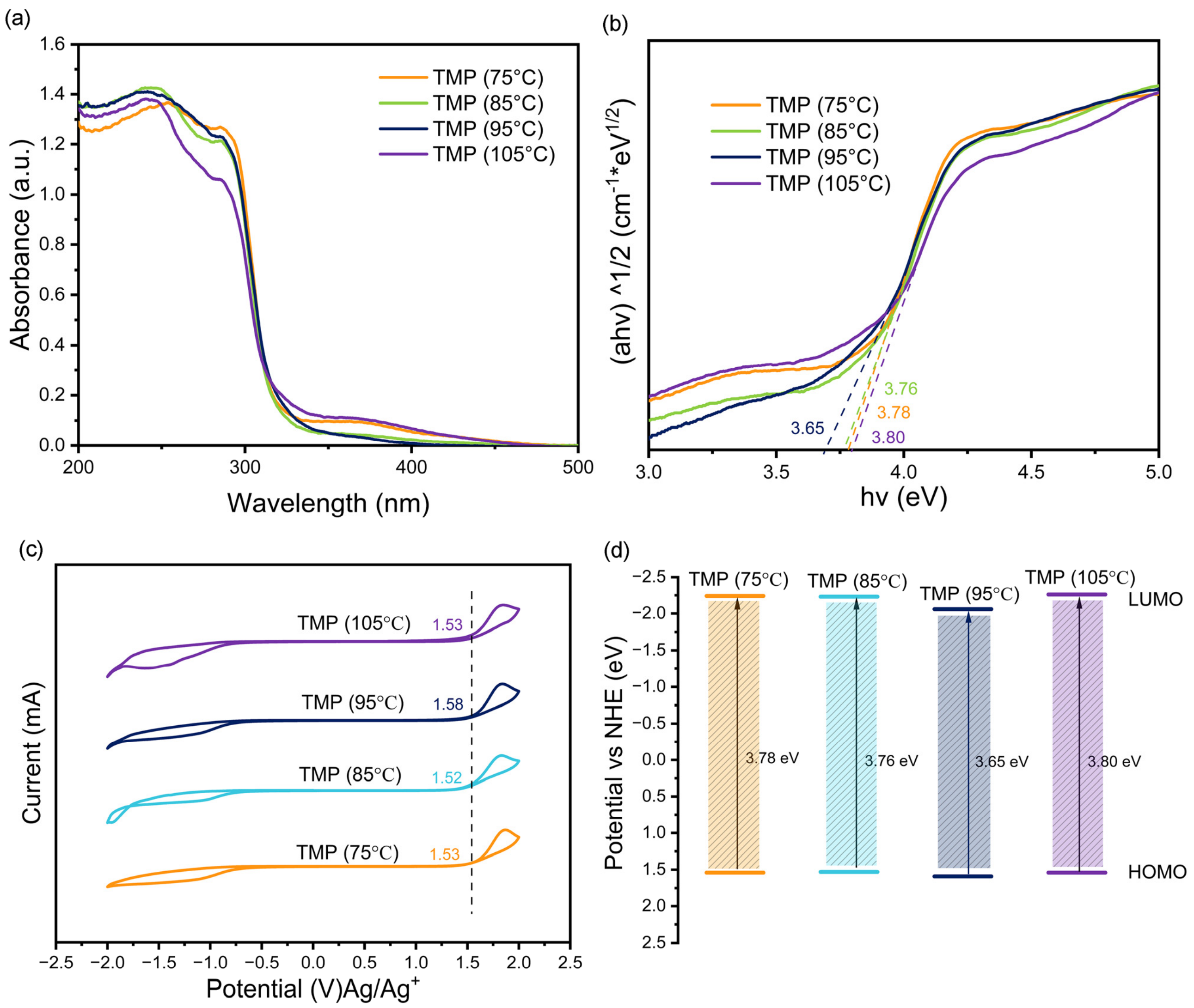

Figure S2. Different reaction temperatures of M and TMC: (**a**) UV-Vis DRS of the TMP; (**b**) Diagram of the Kubelka-Munk function of the TMP versus the absorbed light energy (band gap width); (**c**) Cyclic voltammogram (CV) of TMP; (**d**) Location of TMP HOMO and LUMO.

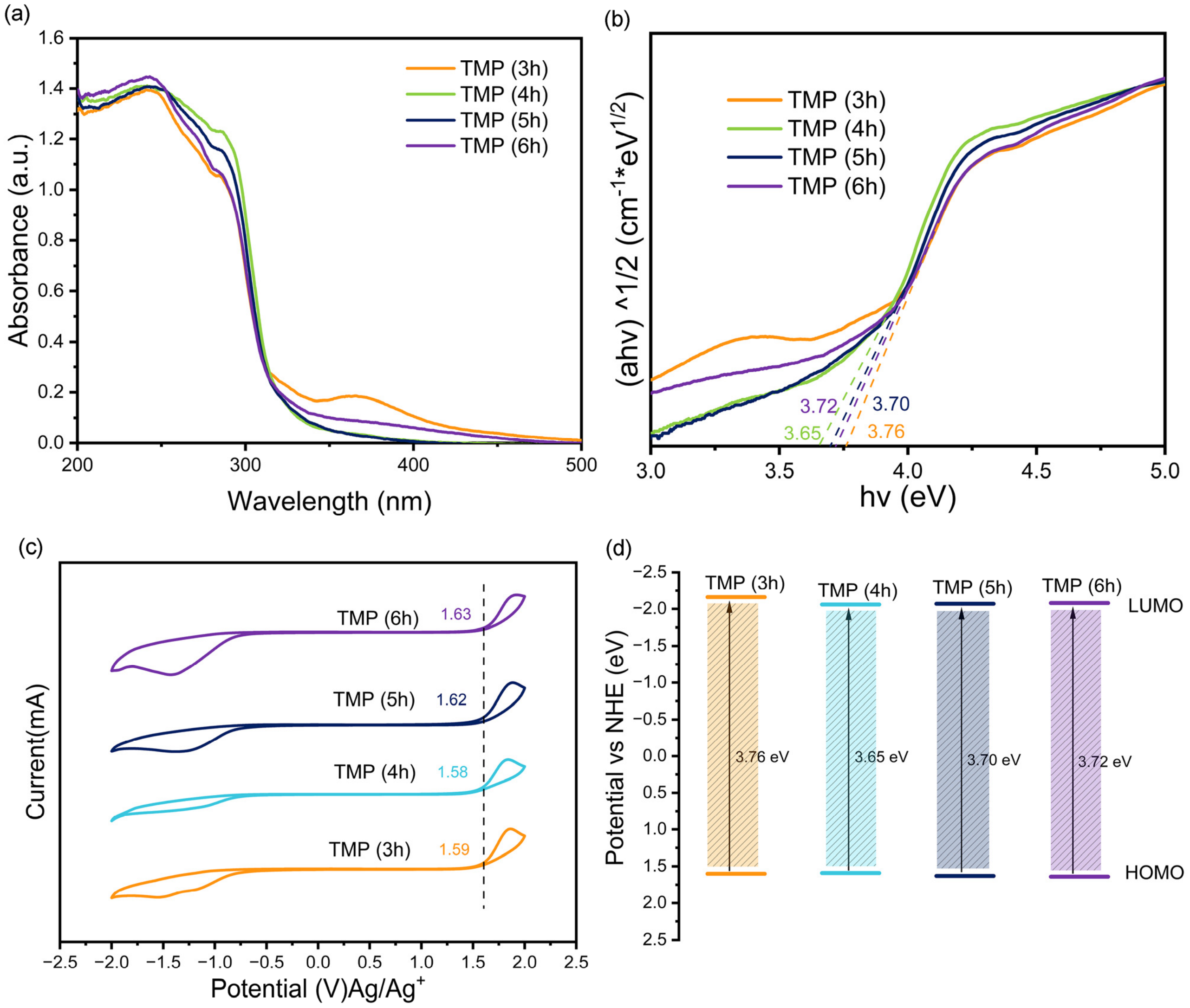

Figure S3. Different reaction time of M and TMC: (**a**) UV-Vis DRS of the TMP; (**b**) Diagram of the Kubelka-Munk function of the TMP versus the absorbed light energy (band gap width); (**c**) Cyclic voltammogram (CV) of TMP; (**d**) Location of TMP HOMO and LUMO.

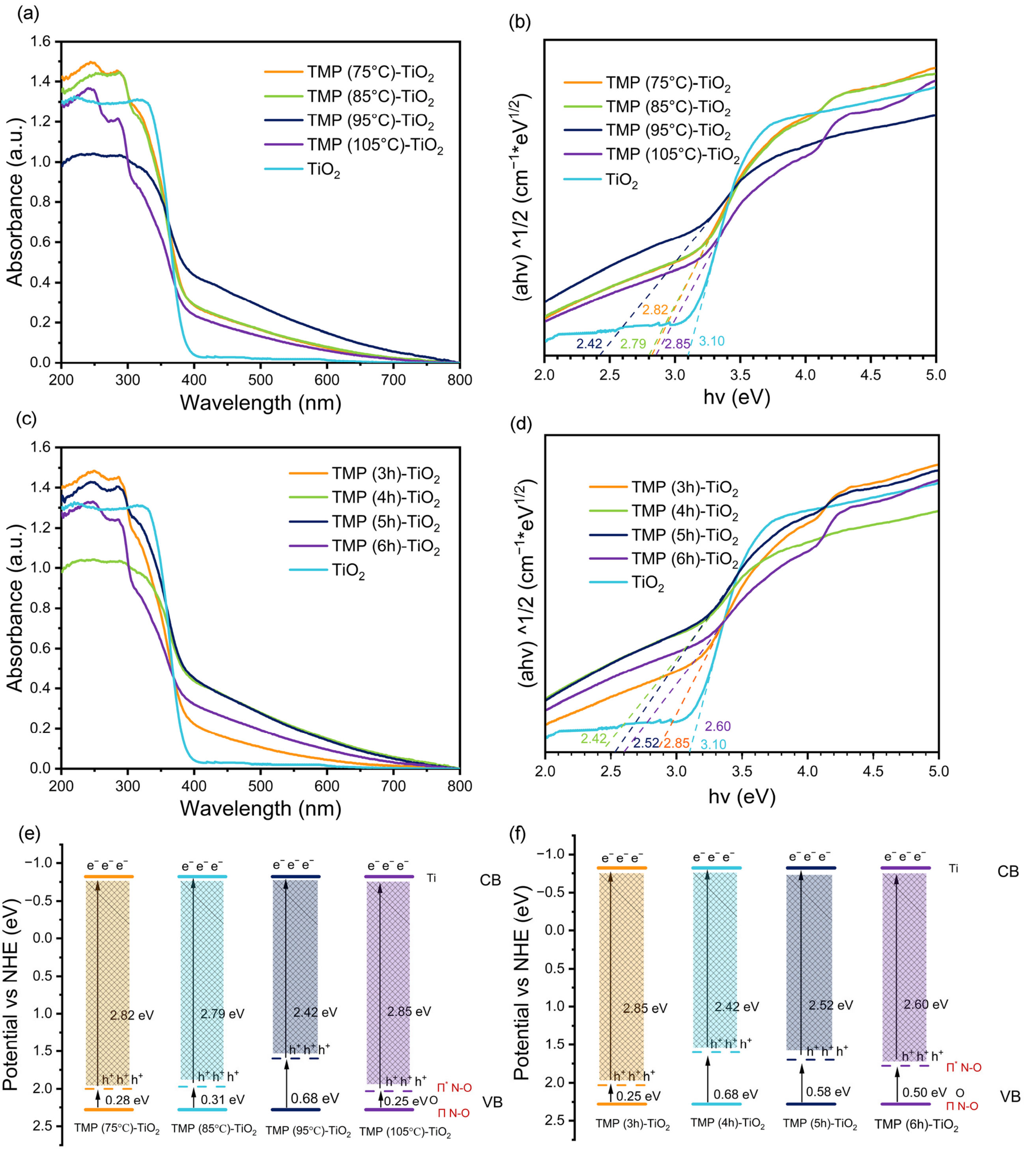

Figure S4. Different reaction temperatures of M and TMC: (**a**) UV-Vis DRS of the TMP-TiO_2; (**b**) Diagram of the Kubelka-Munk function of the TMP-TiO_2 versus the absorbed light energy (band gap width); Different reaction time of M and TMC: (**c**) UV-Vis DRS of the TMP-TiO_2; (**d**) Diagram of the Kubelka-Munk function of the TMP-TiO_2 versus the absorbed light energy (band gap width); Different reaction temperatures of M and TMC: (**e**) Location of TMP-TiO_2 conduction band (CB) and valence band (VB); Different reaction time of M and TMC: (**f**) Location of TMP-TiO_2 conduction band (CB) and valence band (VB).

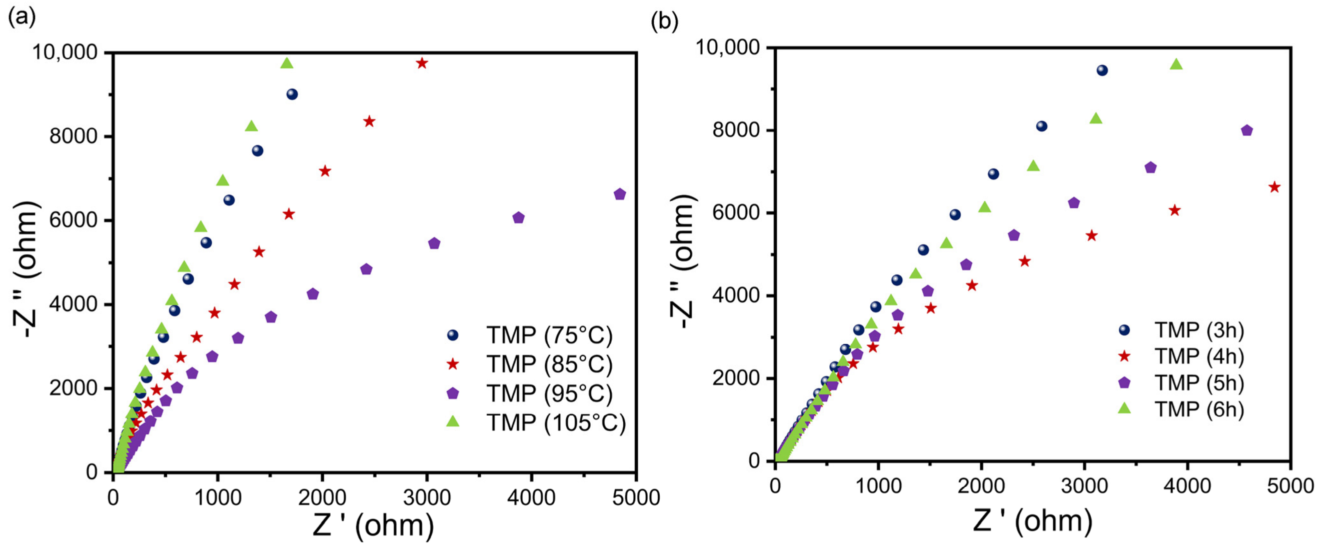

Figure S5. (a) EIS diagram of TMP by varying the reaction temperature of M and TMC; **(b)** EIS diagram of TMP by varying the the reaction time of M and TMC.

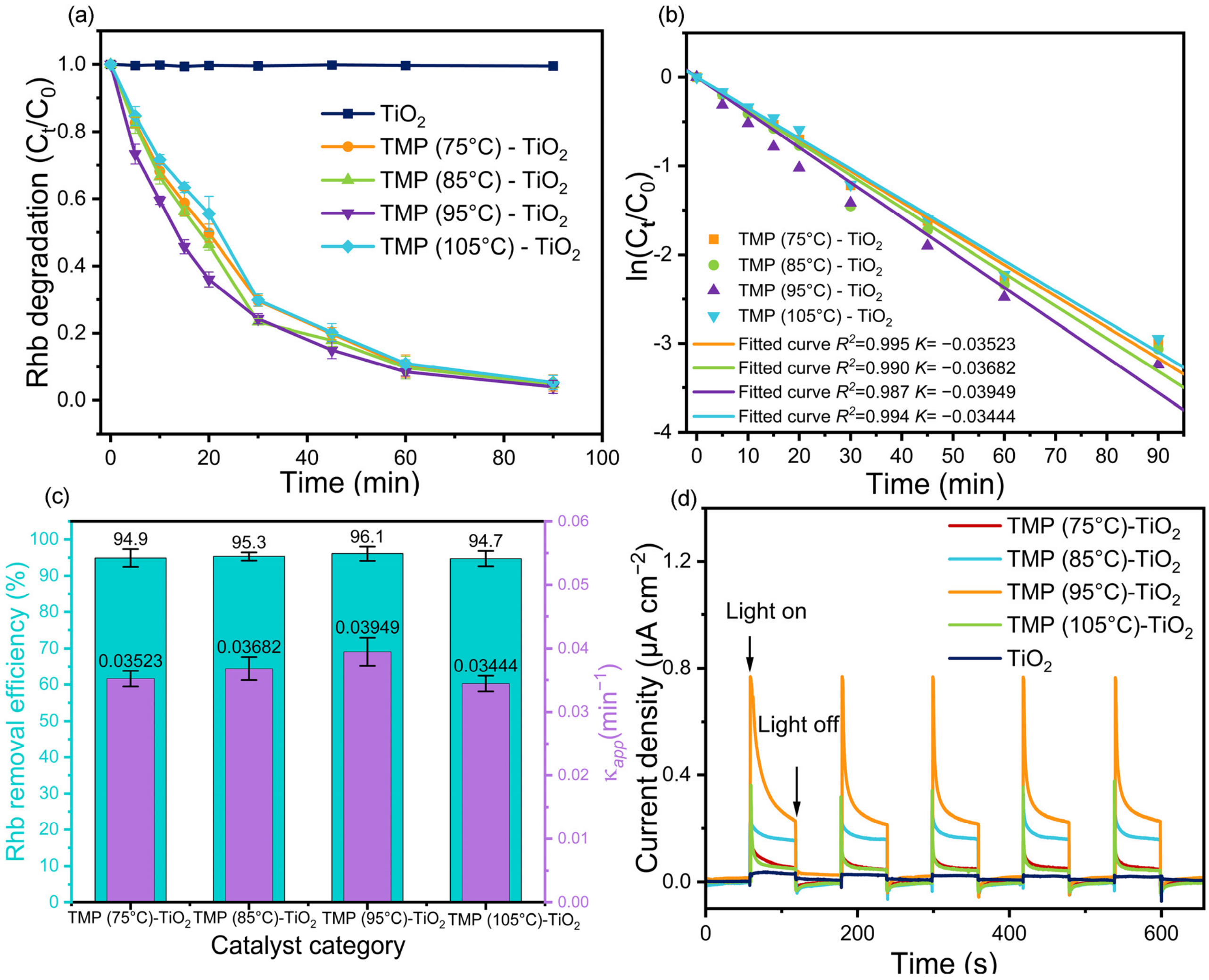

Figure S6. Different reaction temperatures of M and TMC: (**a**) Degradation of RhB; (**b**) Hinshelwood plot for studying the kinetic of the process; (**c**) Removal rate of RhB and value of the linear fit κ_{app}; (**d**) Photocurrent response diagram.

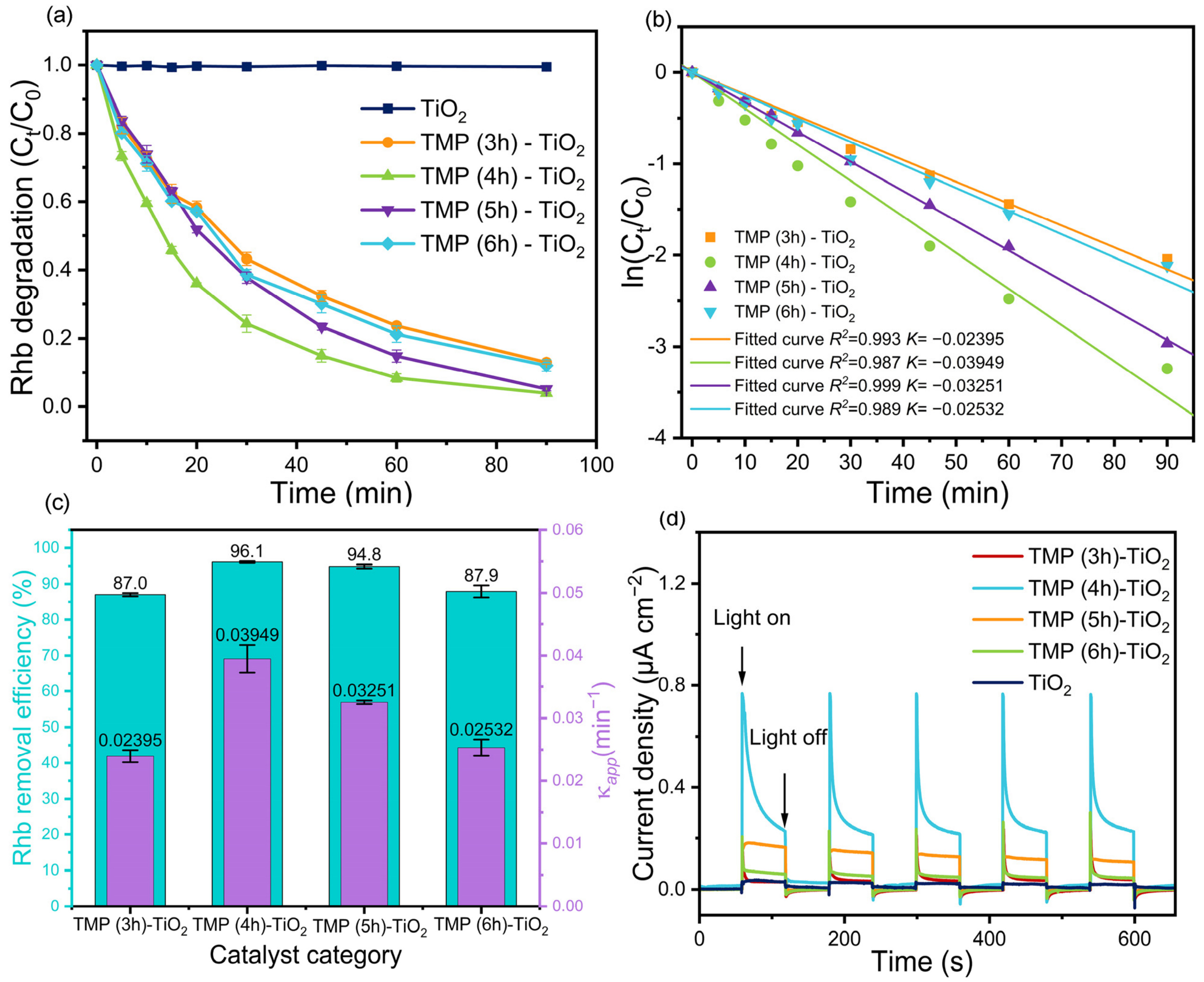

Figure S7. Different reaction time of M and TMC: (**a**) Degradation of RhB; (**b**) Hinshelwood plot for studying the kinetic of the process; (**c**) Removal rate of RhB and value of the linear fit κ_{app}; (**d**) Photocurrent response diagram.

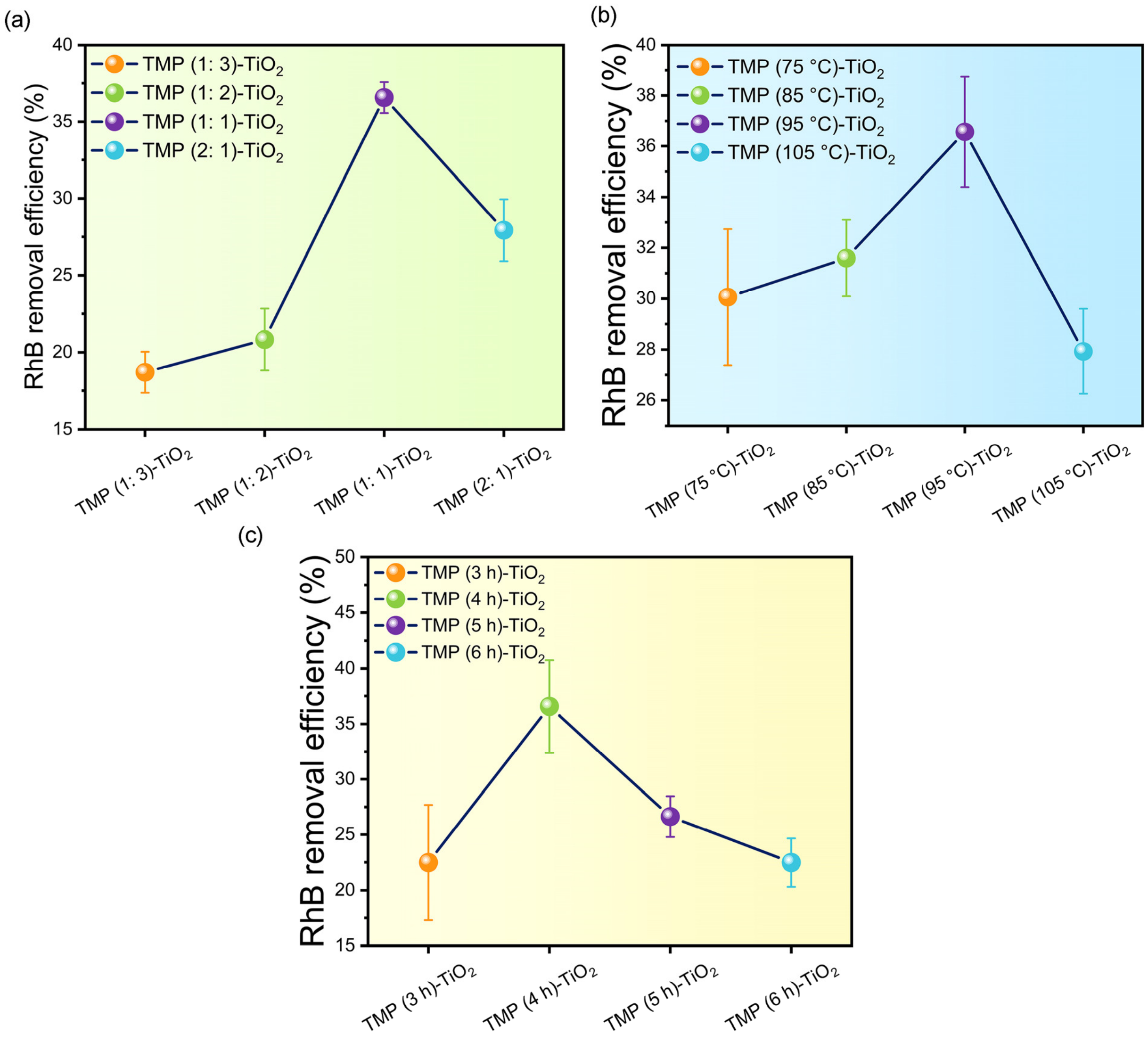

Figure S8. The RhB removal efficiency (%) in adsorption processes (dark condition): (**a**) Different reaction ratios of M and TMC; (**b**) Different reaction temperatures of M and TMC; (**c**) Different reaction time of M and TMC.

Table S2. Comparison of photocatalytic activity of this work with references.

**Material**	**Light source**	**RhB Degradation (%)**	**Experiment conditions**			**Ref.**
			**RhB concentration**	**Catalyst dosage**	**Irradiation time**	
TMP (1: 1)-TiO_2	Visible, 300 W xenon lamp	96.1%	20 mg/L	1 mg/mL	90 min	This work
MS@TiO_2@PPy	Visible, 300 W xenon lamp	90%	10 mg/L	1 mg/mL	90 min	[1]
TiO_2/g-C_3N_4	Visible, 300 W xenon lamp	98.1%	13.3 mg/L	1 mg/mL	105 min	[2]
PVA/TiO_2/CDs	Visible, 300 W xenon lamp	~ 56%	2 mg/L	2 mg/mL	120 min	[3]

Polypyrrole/TiO_2	Simulated solar light	97%	10 mg/L	1 mg/mL	8 h	[4]
PANI-TiO_2/rGO	Visible, 300 W xenon lamp	90.5%	4.79 mg/L	0.5 mg/mL	90 min	[5]
ZnCPP-Fullerol@TiO_2	Visible, 300 W xenon lamp	94.7%	0.025 mg/L	1.14 mg/mL	150 min	[6]

References

1. Yan, S.; Li, Y.; Xie, F.; Wu, J.; Jia, X.; Yang, J.; Song, H.; Zhang, Z. Environmentally safe and porous MS@ TiO_2@ PPy monoliths with superior visible-light photocatalytic properties for rapid oil–water separation and water purification. *ACS Sustainable Chemistry & Engineering* **2020,** 8, (13), 5347-5359.
2. Cui, L.; Liu, S.; Wang, F.; Li, J.; Song, Y.; Sheng, Y.; Zou, H. Growth of uniform g-C_3N_4 shells on 1D TiO_2 nanofibers via vapor deposition approach with enhanced visible light photocatalytic activity. *Journal of Alloys and Compounds* **2020,** 826, 154001.
3. Eskalen, H.; Yaykaşlı, H.; Kavgacı, M.; Kayış, A. Investigating the PVA/TiO_2/CDs polymer nanocomposites: effect of carbon dots for photocatalytic degradation of Rhodamine B. *Journal of Materials Science: Materials in Electronics* **2022,** 33, (7), 4643-4658.
4. Gao, F.; Hou, X.; Wang, A.; Chu, G.; Wu, W.; Chen, J.; Zou, H. Preparation of polypyrrole/TiO_2 nanocomposites with enhanced photocatalytic performance. *Particuology* **2016,** 26, 73-78.
5. Ma, J.; Dai, J.; Duan, Y.; Zhang, J.; Qiang, L.; Xue, J. Fabrication of PANI-TiO_2/rGO hybrid composites for enhanced photocatalysis of pollutant removal and hydrogen production. *Renewable Energy* **2020,** 156, 1008-1018.
6. Wu, Z.-Y.; Xu, Y.-J.; Huang, L.-J.; Zhang, Q.-X.; Tang, D.-L. Fullerene-cored star-shaped polyporphyrin-incorporated TiO_2 as photocatalysts for the enhanced degradation of rhodamine B. *Journal of Environmental Chemical Engineering* **2021,** 9, (5), 106142.
